# Model to personalize mobiles applications according to the gamification user types for health behavioral change

**DOI:** 10.1177/20552076251393402

**Published:** 2025-11-03

**Authors:** Laëtitia Gosetto, Gilles Falquet, Fréderic Ehrler

**Affiliations:** 1 Geneva School of Economics and Management, 27212 University of Geneva, Geneva, Switzerland; 2 Division of Medical Information Sciences, 27230 Geneva University Hospitals, Geneva, Switzerland; 3 Direction of Digital Transformation and Augmented Intelligence, 27230 Geneva University Hospitals, Geneva, Switzerland

**Keywords:** mHealth, behavior change technique, personalization, gamification, Hexad Scale

## Abstract

**Background:**

Adopting healthy habits improves longevity and well-being. Mobile health (mHealth) apps support such behaviors, with over 35,000 available as of 2018. Personalization and gamification are recognized as effective strategies to enhance user engagement and behavioral outcomes in mHealth applications.

**Objectives:**

This cross-sectional study explores links between user typologies (Hexad Scale) and preferences for 15 game and behavior change mechanisms.

**Methods:**

A preference matrix, derived from the literature, was tested on data from 214 respondents (*M*_age_ = 29.42; 118 women, 89 men, 5 other). Demographics and Hexad-based user typologies were recorded. Participants selected their top five mechanisms from 15 randomized mockups with brief descriptions. Logistic and ordinal logistic regressions, with Bonferroni correction, were used to assess associations.

**Results:**

Significant associations were observed for five mechanisms. Philanthropists were less likely to prefer collection (OR = 0.77, *p* < 0.01), whereas players favored it (OR = 1.11, *p* < 0.05) and showed strong preferences for rewards (OR = 1.39, *p* < 0.01) and, to a lesser extent, self-monitoring (OR = 0.88, *p* < 0.05). Socializers preferred cooperation (OR = 1.14, *p* < 0.01) but were less inclined toward demonstration of behavior (OR = 0.92, *p* < 0.05). Free spirits favored demonstration of behavior (OR = 1.25, *p* < 0.01), while achievers were less likely to prefer it (OR = 0.86, *p* < 0.05). Four mechanisms, self-monitoring, progression, challenge, and quest were selected by over 50% of participants.

**Conclusion:**

This study validated the preference matrix, highlighting four mechanisms, self-monitoring, progression, challenge, and quest, as broadly appealing across user profiles for mHealth design. Three novel profile–mechanism associations were identified, refining the model and underscoring the need for replication with a more diverse sample.

## Introduction

Embracing healthy lifestyle habits has been shown to extend life expectancy. In particular, following a balanced diet, maintaining a healthy weight, and engaging in regular physical activity are linked to lower mortality rates. Researchers have found that these behaviors contributes to increased longevity.^1,2^

The number of health apps aimed at supporting individuals in adopting healthier habits has been growing every year, with new apps regularly entering the market. By 2018, over 35,000 health apps were available for download.^3^ Smartphone apps have opened new pathways for individuals to engage in health-focused behaviors, providing instant access to health information, medication reminders, and progress-tracking tools that collectively promote healthier lifestyles.^4–7^

Various tools have been created to evaluate the quality of mobile health (mHealth) applications, such as the Mobile App Rating Scale^8^ and the App Behavior Change Scale.^9^ A common criterion across these evaluation frameworks is the emphasis on personalization. This aspect is considered essential, especially in the context of designing apps aimed at promoting behavioral change. For instance, one study has demonstrated that health messages customized using individual-specific factors (e.g., age, beliefs, health status) were significantly more effective than non-tailored messages in attracting attention, facilitating cognitive processing, and enhancing memory retention. Such personalized content was also perceived as more personally relevant, which increased the likelihood of interpersonal discussion and incorporation into health-related decisions.^10^

An other strategy used to encourage behavioral change is the inclusion of gamification,^11^ defined as “the application of game design elements in non-game contexts”.^12^ Notably, Lister et al. found that 52% of reviewed health apps included at least one gamification feature.^13^ Gamification can be effective for a variety of healthcare issues, such as medication adherence,^14^ behavior change for diabetics patients,^15^ self-efficacy, nursing education,^16^ and motivation to quit smoking.^17^ Indeed, gamification has a positive impact on time spent on the app,^18^ on behavior, user experience and a positive influence on health and well-being.^19^ Therefore a review by Klock et al. addressed the issue of personalization, but in the context of user experience and user interface design with gamification.^12^

### Linking personality to game mechanics

Personalization refers to the adaptation of mechanisms to the user's profile, whereas customization allows the user to actively choose the mechanisms they prefer. A common strategy for personalization is to use player or user typologies to identify individual preferences. A previous study^20^ reviewed the literature to identify associations between user typologies and specific mechanisms that can be integrated into mHealth applications. Based on these insights, a preference matrix was developed, mapping preferred mechanisms to user profiles. This matrix serves as a tool to support the personalization of mhealth apps aimed at promoting behavior change. By aligning app features with user characteristics, developers can enhance engagement and effectiveness. For example, for users identified as socialiser based on the Hexad Scale model, it would be beneficial to emphasize features that encourage cooperation.^21^ The preference matrix includes 15 mechanisms linked to players personality profile. Regarding the user typologies, we utilized one of the most prevalent classification systems for individuals, namely the Hexad Scale.^12^^21–28^

### Gamification user typologies: Hexad Scale

Multiple models have been proposed in the literature to identify individual preferences for game mechanisms, such as the BrainHex model,^29^ Bartle's taxonomy,^30^ and Yee's player motivations.^31^ One of the most widely utilized instruments included on the preference matrix for the assessment of player typologies is the Hexad Scale Model. The Hexad Scale model characterizes an individual's player typologies based on six distinct typologies,^27^ as illustrated in Table 1. The scale assigns a score to each dimension, reflecting the extent to which each typology is present in a given user.

**Table 1.** Definition of the Hexad Scale player typologies.

User types	Definition
Philantropists	Motivated by purpose. Altruistic and willing to give without expecting a reward.
Socialisers	Motivated by relatedness. Want to interact with others and create social connections.
Free Spirits	Motivated by autonomy. Freedom to express themselves and act without external control. Like to create and explore within a system.
Achievers	Motivated by competence. Seek to progress within a system by completing tasks or prove themselves by tackling difficult challenges.
Players	Motivated by extrinsic rewards. Will do whatever to earn a reward within a system, independently of the type of the activity.
Disruptors	Motivated by triggering of change. Tend to disrupt the system either directly or through others to force negative or positive changes. They like to test the system's boundaries and try to push further.

### Selection of the 15 mechanisms

A review of the literature identified 15 processes that have been linked to user typologies,^20^ based on the idea that people with different profiles have varying preferences regarding elements of mHealth applications that attempt to facilitate behavioral change. The word “mechanism” in our case refers to all of the elements that might be included in a mHealth application that aims to modify behavior. We divided the mechanisms into two groups: those associated with gaming components and those associated with behavior change strategies. See Table 2 for more information and a definition of the mechanisms.

**Table 2.** List of mechanisms with their definition.

Mechanism	Definition
**BCT mechanisms**	
Prompt and cues	Usually a message delivered to the user to prompt or recall a behavior at a specified time, with the app or user defining when the message should be sent ^32^
Demonstration of the behavior	Enables users to observe the cause-and-effect linkage of their behavior, such as seeing a simulation of their bodies after a diet ^33^
Self-monitoring	Users can track their behaviors, providing information on both past and current activities ^33^
Punishment	Virtually penalizes the user for not performing the desired behavior or reaching their goal ^34^
Social comparison	Highlight others’ performance to enable comparison with one's own^35^
Social support	Enables communication between users, such as through chat or sharing activities with other users ^12^.
**Game elements**	
Progression	Users can track their progression with steps through the system's purpose over time, visualized with mechanisms like stars or flags along a path ^33^.
Competition	Users can compete to accomplish the desired behavior ^33^.
Cooperation	Users collaborate to achieve a shared objective ^33^.
Collection	Allows users to gather virtual objects. Groups of rewards or badges to earn ^36^
Rewards	Virtual rewards offered to users for engaging in the target behavior ^33^.
Quest	Users can enter or define the objectives targeted for the activity they will perform ^32^.
Challenge	Presents various situations that require effort from the user to be completed ^12^ (e.g., accomplishing 3 h of physical activity per week).
Avatar	Allows users to share their data in the system without revealing their name ^12^.
**App mechanism**	
Customization	In contrast to personalization, which involves adjusting automatically the system to the user, customization refers to the user's ability to modify the content or functionalities of the mobile application according to their own preferences ^33^. This approach enables users to actively tailor the system based on users’ choices.

### Preferences relations between Hexad Scale player typologies and mechanisms

Based on this literature review, a preference matrix could be generated, which is presented in Table 3, using the preference relations between the mechanisms and Hexad Scale player typologies that were discovered in the literature.^20^ Each cell of the matrix indicates the number of articles that had a preference connection. A preference for a particular mechanism within a certain profile was indicated by a plus sign for the majority of the potential preference relations, which were positive (67%, 60/90). Negative relations, which make up 6% (5/90) of all possible relations, show an aversion to a mechanism (shown by a minus sign in Table 3). It should be mentioned that only 67% (60/90) of the possible linkages were discovered during the literature review, therefore the relation matrix is still incomplete.

**Table 3.** Preference relations between gamer profile Hexad Scales and mechanisms (+ represent preference relation and − an aversion relation).

			BCT mechanisms						Game elements								App mechanism
			Prompts and cues	Demonstration of the bahavior	Self-monitoring	Punishment	Social comparison	Social support	Progression	Competition	Cooperation	Collection	Rewards	Quest	Challenge	Avatar	Customization
Gamer profile	Hexad Scale	Disruptor			+ -		+		++ -	+++			+		++	+	++
		Philanthropist		+				+	++		++	++	+	+	+		+
		Socialiser		+	+ -	+	+++	++++	++ -	++++	+++++		++	+	++	+	+
		Player				+	+++	+	++++	+++++	++	++++	++++	+	++++	++	+
		Free spirit						+	+++		+		+	+	++++	+	++
		Achiever					+		++++	+	+	++	+++ -	++	++++	+	+

+ 1 article with a preference relation − 1 article with non-preference relation.

This study aims to corroborate and potentially expand the existing preference matrix (Table 3), which maps relationships between game elements and BCT mechanisms with the Hexad model, by identifying new preference relations through experimental testing.

## Methods

This section comes from our previous published protocol article.^37^

### Ethics approval

This study was approved by the University of Geneva Ethics Commission (CUREG_2021-04-38). Written informed consent was obtained electronically from all participants prior to study initiation. At the beginning of the online questionnaire, participants were presented with a consent section describing the study's objectives, procedures, data handling, and their right to withdraw at any time. Access to the questionnaire was granted only after participants confirmed that they had read and accepted these terms.

### Study design

We conducted a cross-sectional study to test and expand the existing preference matrix (Table 3), which maps relationships between gamification mechanisms and player typologies. This design was chosen to allow simultaneous collection of participants’ user profile data (via the Hexad Scale) and their experimentally assessed preferences for specific mechanisms. The goal was to determine whether the preference patterns identified in our prior scoping review (Table 3) could be corroborated in a controlled, experimental setting and whether new preference relations could be uncovered.

Participants completed an online questionnaire designed in accordance with the Checklist for Reporting Results of Internet E-Surveys.^38^ The survey included two core tasks: (1) completion of the Hexad Scale to assess user typology, and (2) selection and rating of motivational potential for five preferred mechanisms from a set of 15 mockups. These mechanisms were drawn from our previous work and represented diverse engagement strategies in mHealth applications.

#### Outcomes

The primary outcome is the preferred mechanisms given the user typologies.

#### Study population

The target population for this study is individuals aged 18 and above who understands French. The recruitment process was conducted via social media platforms, specifically Facebook and Twitter, targeting students at the University of Geneva.

#### Sample size calculation

As part of a larger study on the Big Five personality traits, the required sample size to identify a significant difference in preference was estimated using a multiple regression power analysis in R (*u* = 3, *f²* = 0.07, α = 0.05, power = 0.90). The variance estimate (202.403) was derived from prior research linking altruism, as measured by the Big Five, to preferences for social network content.^12,22^ Specifically, we used variance data from altruistic participants’ responses (*n* = 46) to a blood donation poster (score range: 0–100).^39^ Based on these parameters, a minimum of 206 participants was determined to be adequate.

#### Procedure

Participants were invited to complete an online survey developed by the research team using Qualtrics software (Qualtrics, Provo, UT) (see Multimedia Appendix 1). The process began with an informed consent. Participants were required to confirm that they had read and understood the form and agreed to its terms before accessing the questionnaire, allowing the researchers to use their responses for the study.

Next, participants provided demographic information and were asked to confirm that they were at least 18 years old to proceed. Those who met the age requirement continued to the main part of the questionnaire, which involved two tasks: (1) completing a Hexad Scale assessment and (2) reviewing 15 proposed mechanisms, selecting their five preferred ones, and rating the extent to which each would motivate them to adopt healthier behaviors on a scale from 0 to 100.

#### Measures and measurement

##### Demographic questions

The participants were requested to provide information regarding their gender, age, occupation, and level of education.

### Profile assessment

#### Hexad Scale profile

To assess participants’ gamification user type, we relied on Hexad Scale created and validated by Tondello.^27^ The internal scale reliability is good with Cronbach's alpha coefficient for each dimension ranging from 0.70 to 0.89.^27^ This scale consists of 24 items, 4 per dimension. Users must rate how well each article describes them on a 7-point Likert scale. For example, there are items such as “I like competitions, where a prize can be won” or “Interacting with others is important to me.” Items are presented in a randomized manner and the score is calculated by adding the scores for each dimension.

### Choice of mechanisms

#### Presentation and selection of the five favorites mechanisms

Mockups representing each of the 15 mechanisms (see Appendix 1) were developed. A detailed description and definition of each mechanism is available in the Multimedia Appendix. To minimize potential bias related to aesthetic preferences, all mockups were designed using a deliberately simple and neutral visual style, limited to a black-and-white palette and basic icons. The order of presentation was randomized for each participant to prevent primacy or recency effects. During the study, participants were asked to select the five mechanisms they found most motivating, based on both the visual mockup and an accompanying brief textual explanation.

#### Motivation score

Participants were asked to rate on a scale of 0 to 100% how motivated these mechanisms made them feel to get back in shape for each of the five mechanisms they had previously selected.

#### Explanation of choice

For each mechanism selected, participants were asked to rate its perceived motivational potential in encouraging healthier behaviors using a scale ranging from 0 to 100. In addition, they were invited to provide an open-ended justification explaining their choices. Mechanisms that were not selected were automatically assigned a score of zero.

#### Analysis

##### Preference matrix validation

In order to perform a comparative analysis of the experimental results and the initial preference matrix (M1), the collected data were also organized into a matrix. The values of the initial matrix (M1) represent the number of preference relations observed in the literature following the scoping review that we conducted previously.^20^ We modified this matrix by assigning a value of −1 to indicate an aversion relation. A value of 0 indicates that a similar number of preferences and aversions were observed in the scoping review, and a null value is assigned when no relations were identified. The second matrix (M2) contains the data collected in this study. The value of the cell represents the score of the participants with the Hexad Scale dimension for the mechanism on the line. In each cell, we calculated a mechanism discriminative score by summing the participants’ scores, divided by the total number of participants:

$$C_{m,b}=\mathrm{round}\left(\;\frac{\sum_{\;p=1}^{n}\;1_{\;p,m}\cdot S_{\;p,b}}{\sum_{\;p=1}^{n}\;1_{\;p,m}}-\frac{\sum_{\;p=1}^{n}\;0_{\;p,m}\cdot S_{\;p,b}}{\sum_{\;p=1}^{n}\;0_{\;p,m}}\right)$$

*C_m,b_* represents the value in the cell corresponding to mechanism m (row) and the Hexad Scale b (column). 1*_p,m_* is an indicator function equal to 1 if participant p has selected mechanism m, and 0 otherwise. 0*_p,m_* is an indicator function equal to 0 if participant p has selected mechanism m, and 1 otherwise. *S_p,b_* is the score of participant p on the Hexad Scale b dimension (4–28). *n* is the total number of individuals.

A third matrix (MC) represents a comparison between the two matrices (M1, M2). The values are computed by the difference on the score of both matrices.

#### Statistical analysis

A logistic regression analysis was conducted to examine the relationship between the mechanisms and the player profile scales. The objective of this analysis was to analyze if the participants’ scores on the Hexad Scale significantly influences the choice of a mechanism. A logistic regression analysis was conducted for each mechanism to ascertain whether the Hexad Scale scores were predictive of the mechanism selection.

Furthermore, a logistic ordinal regression was conducted with the motivation scores of the selected mechanisms as the dependent variables and the Hexad Scale scores as the predictors. The regression was performed for each mechanism motivation score, with the objective of determining whether the scores on the scales predict the mechanism score. To prevent a type I error, the Bonferroni correction was employed for all regression analyses.

## Results

### Demographics data

A total of 214 individuals responded to our questionnaire, including 118 women, 89 men, 5 others, and 2 didn’t answer. The average age was 29.42 years (SD = 10.41). More details are given in Table 4.

**Table 4.** Demographics data.

	*N*	Mean	Standard deviation
Age	214	29.42	10.41
	*N*	%	
Gender	214		
Women	89	41.6	
Men	118	55.1	
Others	7	3.3	
Education Level	211		
Mandatory education	53	24.8	
Bachelor's degree	63	29.4	
Master's degree	80	37.4	
Doctorate	15	7	
Smartphone use	214		
Not confortable	10	4.7	
Confortable	204	95.3	
Already used mHealth	135	63.1	

#### Representativeness of the participants on the Hexad Scale

**Figure 1.**
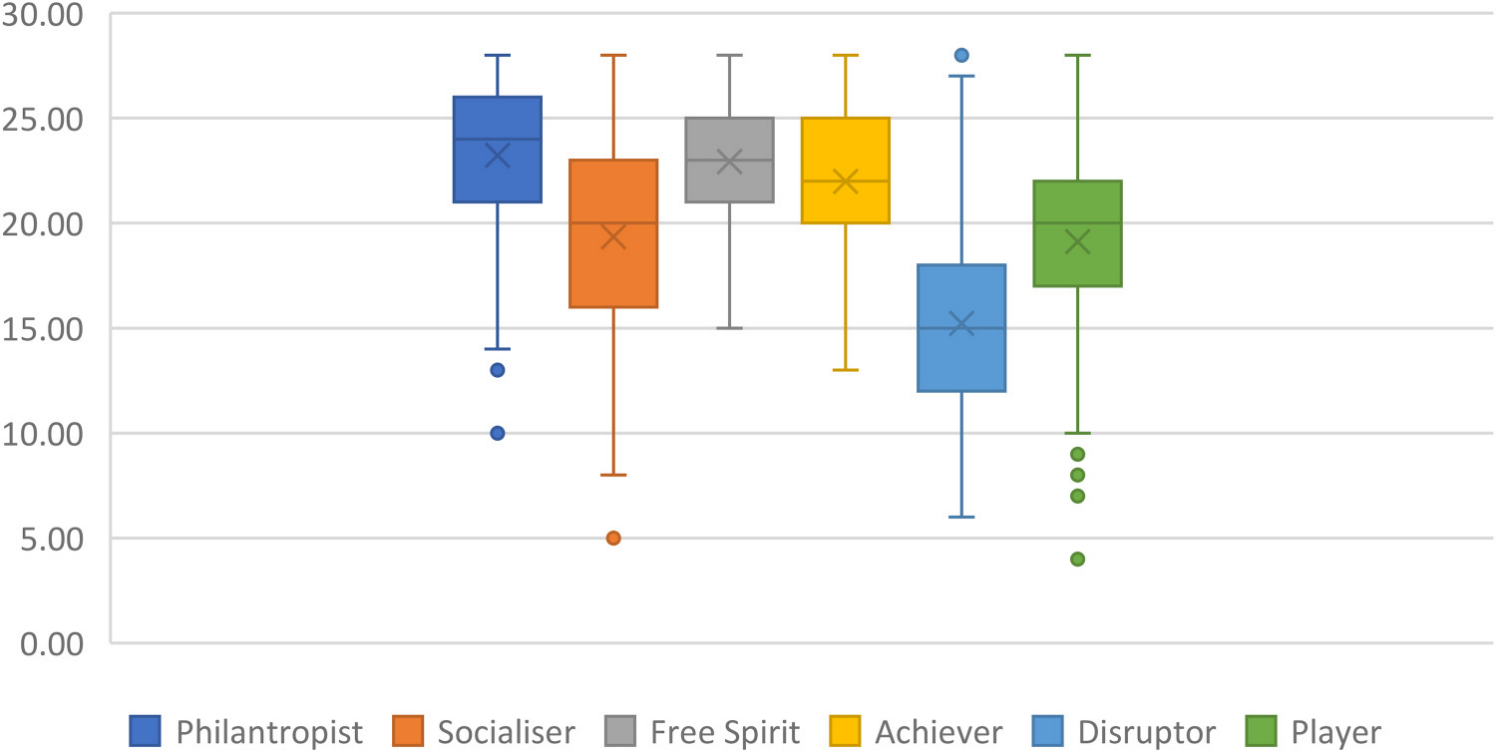
Boxplot of the representativeness of the participants on the Hexad Scale (*N* = 214).

The scores for the Hexad Scale dimensions are recorded on a scale of 4–28. The distribution of the population on the Hexad Scale is shown in Figure 1 (the line represents the median and the cross represents the mean of the population). Figure 1 represents the overall tendency of the sample toward each gamer type dimension, showing whether participants, on average, scored higher or lower on specific motivational orientations captured by the Hexad Scale.

The participants of this study are mainly philantropist (M = 23.23, SD = 24.50), Free spirit (M = 22.93, SD = 24.00), Achiever (M = 21.99, SD = 21.50), Socialiser (M = 19.35, SD = 16), and Player (M = 19.11, SD = 17.50). They are lower Disruptor (M = 15.22, SD = 14.50).

### Characteristics of the participants on the selection for each mechanism

Four mechanisms, self-monitoring, progression, challenge, and quest, were selected by more than half of the participants, indicating a broad, cross-participant preference for these features (Table 5).

**Table 5.** Number of people who selected the mechanism (*N* = 214).

Mechanisms	Number of people who selected the mechanism	Percentage
Self-monitoring	149	69.6
Progression	127	59.3
Challenge	112	52.3
Quests	111	51.9
Cooperation	70	32.7
Demonstration of the behavior	61	28.5
Prompt and cues	53	24.8
Rewards	47	22
Social comparison	44	20.6
Collection	41	19.2
Avatar	29	13.6
Competition	29	13.6
Social support	20	9.3
Punishment	8	3.7

### Comparison between this study results and the preference matrix

**Figure 2.**
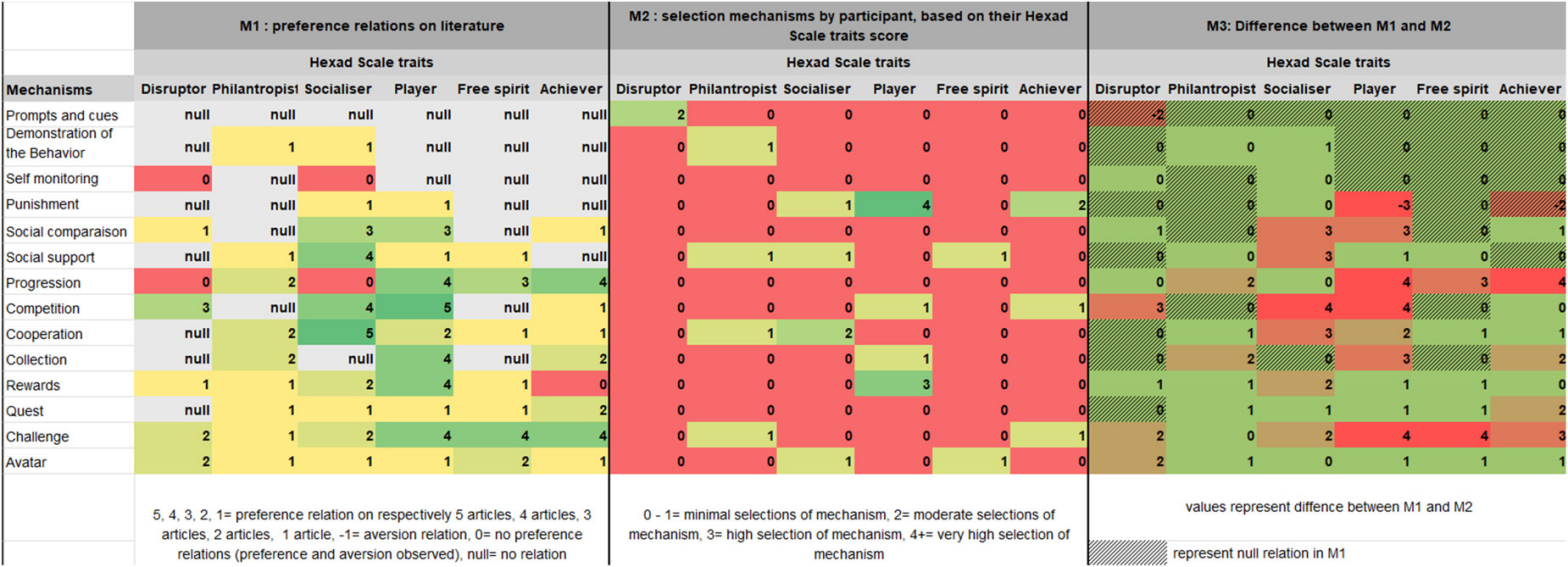
Matrices for the representation of preference relations on literature (M1), of the selection mechanisms by participants based on their Hexad Scale score (M2), and the difference between M1 and M2 (M3).

The initial matrix (M1) presents 60% (50/84) of positive relation, 0% of aversion relation, 6% (5/84) of non-congruent relations (preference and aversion were observed) and 35% (29/84) of missing relations with no evidence found in our review (see M1 on Figure 2). Therefore, it is evident that the initial matrix derived from the scoping review is incomplete, with 35% of relationships remaining unaccounted for and potentially addressable explored through this research. Moreover, the predominance of positive relationships within this matrix highlights a gap in the literature, which has paid limited attention to documenting aversion-based relationships toward specific mechanisms.

The matrix of the selection mechanisms by participant of this study according on their Hexad Scale traits score (M2) (M2) presents 1% (1/84) of very high mechanism discriminative score (>4), 1% (1/84) of high selection mechanism (3), 4% (3/84) of medium selection mechanism (2), 92% (77/84) of low selection mechanisms (0–1) (see M2 on Figure 2). Consequently, we observe a minimal prevalence of high and medium selection mechanisms, accounting for a mere 6% of the total. The preponderance is predominantly characterized by weak selection mechanisms, which account for an overwhelming 92% of the observed cases.

In the matrix of presenting the difference between M1 and M2, the higher the value, the greater the difference between the 2 matrices. We observe 46% (39/84) similar value in the 2 matrices (0), 23% (19/84) minimal difference of one, 13% (11/84) moderate difference of two, and 18% (15/84) significant difference of three and more (see M3 on Figure 2). We observe a 46% similarity between the two matrices, which is close to half and therefore indicates a moderate level of overlap. Therefore, it is expected that the results of our study will partially align with those of matrix M1, which reflects the outcomes of the scoping review. Additionally, the objective is to identify novel and substantial results that are not present in matrix M1, given the observed discrepancies between the two matrices, which account for 54% of the difference matrix M3.

### Preferred mechanisms and Hexad Scale

The full model containing all predictors was statistically significant for five selections of mechanism.

#### Mechanism collection

The full model was statistically significant, χ^2^ (6, *N* = 205) = 22.16, *p* < 0.05, indicating that the model was able to distinguish between respondants who selected and did not select the mechanism collection. The model as a whole explained between 10% (Cox and Snell R square) and 17% (Nagelkerke R squared) of the variance in the selection of the mechanism collection, and correctly classified 82.9% of cases. As shows in Table 6, two of the independent variables made a unique statistically significant contribution to the model (philanthropist and player). The strongest predictors of selecting the mechanism collection were player with an odds ratio of 1.11. The odds ratio of .76 for philantropist was less than 1, indicating that the respondents with a high level of philantropist were over .76 times less likely to select this mechanism.

**Table 6.** Logistic regression predicting likelihood of selecting the mechanism collection.

	B	S.E	Wald	*df*	*p*	Odds ratio	95% CI for odds ratio	
Philantropist	−.271	.075	13.134	1	**<0**.**01**	.763	.659	.883
Socialiser	.071	.052	1.827	1	0.177	1.073	.969	1.189
Free Spirit	.096	.084	1.290	1	0.256	1.101	.933	1.298
Achiever	−.049	.067	.548	1	0.459	.952	.836	1.085
Disruptor	−.026	.053	.244	1	0.621	.974	.878	1.081
Player	.106	.052	4.092	1	**<0**.**05**	1.112	1.003	1.232
Constant	.345	1.836	.035	1	0.851	1.412		

#### Mechanism cooperation

The full model was statistically significant, χ^2^ (6, *N* = 209) = 17.87, *p* < 0.01, indicating that the model was able to distinguish between respondants who selected and did not select the mechanism cooperation. The model as a whole explained between 8% (Cox and Snell R square) and 11% (Nagelkerke R squared) of the variance in the selection of the mechanism cooperation, and correctly classified 67.5% of cases. As shows in Table 7, one of the independent variables made a unique statistically significant contribution to the model (socializer). The strongest predictors of selecting the mechanism cooperation were socialiser with an odds ratio of 1.1.

**Table 7.** Logistic regression predicting likelihood of selecting the mechanism cooperation.

	B	S.E	Wald	*df*	*p*	Odds ratio	95% CI for odds ratio	
Philantropist	.070	.061	1.314	1	0.252	1.073	.951	1.210
Socialiser	.128	.042	9.365	1	**<0**.**01**	1.137	1.047	1.234
Free Spirit	−.125	.068	3.424	1	0.064	.882	.773	1.007
Achiever	.051	.052	.964	1	0.326	1.052	.951	1.165
Disruptor	−.020	.039	.250	1	0.617	.981	.908	1.059
Player	−.050	.037	1.761	1	0.185	.952	.884	1.024
Constant	−1.949	1.442	1.827	1	0.176	.142		

The full model was statistically significant, χ^2^ (6, *N* = 211) = 13.01, *p* < 0.05, indicating that the model was able to distinguish between participants’ scores for motivation to get fit using the cooperation mechanism. The model as a whole explained between 6% (Cox and Snell R square) and 7% (Nagelkerke R squared) of the variance in the selection of the mechanism cooperation. As shown in Table 8, independent variables socialiser made a unique statistically significant contribution to the model. The predictors Socialiser of scoring the mechanism cooperation were with an odds ratio of 1.10.

**Table 8.** Logistic regression predicting likelihood of score of the mechanism cooperation.

	B	S.E	Wald	*df*	*p*	Odds ratio	95% CI for odds ratio	
Philantropist	.062	.057	1.161	1	0.281	1.064	−.051	.175
Socialiser	.095	.038	6.346	1	**<0**.**05**	1.100	.021	.170
Free Spirit	−.075	.063	1.408	1	0.235	.928	−.199	.049
Achiever	.031	.049	.411	1	0.522	1.032	−.064	.127
Disruptor	−.029	.037	.603	1	0.437	.972	−.102	.044
Player	−.050	.035	2.000	1	0.157	.951	−.120	.019

#### Mechanism rewards

The full model was statistically significant, χ^2^ (6, *N* = 208) = 38.67, *p* < 0.01, indicating that the model was able to distinguish between respondants who selected and did not select the mechanism rewards. The model as a whole explained between 17% (Cox and Snell R square) and 26% (Nagelkerke R squared) of the variance in the selection of the mechanism cooperation, and correctly classified 78.8% of cases. As shows in Table 9, independent variables Player made a unique statistically significant contribution to the model. The predictors Player of scoring the mechanism rewards were with an odds ratio of 1.39.

**Table 9.** Logistic regression predicting likelihood of selecting the mechanism rewards.

	B	S.E	Wald	*df*	*p*	Odds ratio	95% CI for odds ratio	
Philantropist	.047	.066	.503	1	0.478	1.048	.921	1.192
Socialiser	−.052	.048	1.152	1	0.283	.950	.864	1.044
Free Spirit	−.121	.082	2.173	1	0.140	.886	.754	1.041
Achiever	−.081	.064	1.590	1	0.207	.922	.814	1.046
Disruptor	.051	.051	1.027	1	0.311	1.053	.953	1.163
Player	.329	.065	25.932	1	**<0**.**01**	1.390	1.225	1.578
Constant	-4.362	1.800	5.870	1	0.015	.013		

The full model was statistically significant, χ^2^ (6, *N* = 211) = 31.65, *p* < 0.01, indicating that the model was able to distinguish between participants’ scores for motivation to get fit using the rewards mechanism. The model as a whole explained between 14% (Cox and Snell R square) and 18% (Nagelkerke R squared) of the variance in the selection of the mechanism rewards. As shown in Table 10, independent variables Player made a unique statistically significant contribution to the model. The predictors Player of scoring the mechanism cooperation were with an odds ratio of 1.31.

**Table 10.** Logistic regression predicting likelihood of score of the mechanism rewards.

	B	S.E	Wald	*df*	*p*	Odds ratio	95% CI for odds ratio	
Philantropist	.003	.060	.002	1	0.961	1.003	−.115	.121
Socialiser	−.033	.045	.550	1	0.458	.967	−.121	.054
Free Spirit	−.050	.075	.435	1	0.510	.952	−.197	.098
Achiever	−.087	.059	2.168	1	0.141	.916	−.203	.029
Disruptor	.023	.047	.245	1	0.621	1.024	−.069	.116
Player	.273	.056	23.586	1	**<0**.**01**	1.314	.163	.383

#### Mechanism self-monitoring

The full model was statistically significant, χ^2^ (6, *N* = 209) = 14.89, *p* < 0.05, indicating that the model was able to distinguish between respondants who selected and did not select the mechanism self-monitoring. The model as a whole explained between 6.9% (Cox and Snell R square) and 9.8% (Nagelkerke R squared) of the variance in the selection of the mechanism cooperation, and correctly classified 70.3% of cases. As shows in Table 11, independent variables Player made a unique statistically significant contribution to the model. The predictors Player of scoring the mechanism self-monitoring were with an odds ratio of .88.

**Table 11.** Logistic regression predicting likelihood of selecting the mechanism self-monitoring.

	B	S.E	Wald	*df*	*p*	Odds ratio	95% CI for odds ratio	
Philantropist	−.016	.056	.079	1	0.779	.984	.881	1.099
Socialiser	−.030	.040	.569	1	0.451	.971	.898	1.049
Free Spirit	.032	.068	.223	1	0.637	1.033	.903	1.181
Achiever	.041	.053	.596	1	0.440	1.041	.940	1.154
Disruptor	−.072	.041	3.061	1	0.080	.931	.859	1.009
Player	−.126	.042	8.989	1	**<0**.**05**	.881	.811	.957
Constant	3.764	1.500	6.302	1	0.012	43.133		

#### Mechanism demonstration of the behavior

The full model was statistically significant, χ^2^ (6, *N* = 208) = 23.01, *p* < 0.01, indicating that the model was able to distinguish between respondants who selected and did not select the mechanism demonstration of the behavior. The model as a whole explained between 11% (Cox and Snell R square) and 15% (Nagelkerke R squared) of the variance in the selection of the mechanism demonstration of the behavior, and correctly classified 73.1% of cases. As shows in Table 12, three of the independent variables made a statistically significant contribution to the model (socializer, free spirit and achiever). The strongest predictors of selecting the mechanism demonstration of the behavior were socialiser with an odds ratio of .91, free spirit with an odds ratio of 1.25, and achiever with an odds ratio of .86. The independent variable philanthropist made a statically tendential contribution with an odds ratio of 1.13.

**Table 12.** Logistic regression predicting likelihood of selecting the mechanism demonstration of the behavior.

	B	S.E	Wald	*df*	*p*	Odds ratio	95% CI for odds ratio	
Philantropist	.122	.064	3.594	1	0.058	1.130	.996	1.282
Socialiser	−.088	.038	5.275	1	**<0**.**05**	.915	.849	.987
Free Spirit	.224	.075	8.902	1	**<0**.**01**	1.251	1.080	1.449
Achiever	−.155	.057	7.497	1	**<0**.**05**	.856	.766	.957
Disruptor	−.034	.041	.692	1	0.405	.967	.892	1.047
Player	−.061	.040	2.273	1	0.132	.941	.870	1.018
Constant	−2.342	1.576	2.209	1	0.137	.096		

The full model was statistically tendential, χ^2^ (6, *N* = 211) = 12.25, *p* = 0.06, indicating that the model was able to distinguish between participants’ scores for motivation to get fit using the demonstration of the behavior mechanism. The model as a whole explained between 6% (Cox and Snell R square) and 7% (Nagelkerke R squared) of the variance in the selection of the mechanism demonstration of the behavior. As shown in Table 13, independent variables Free Spirit made a unique statistically significant contribution to the model. The predictors Free Spirit of scoring the mechanism cooperation were with an odds ratio of 1.17.

**Table 13.** Logistic regression predicting likelihood of score of the mechanism demonstration of the behavior.

	B	S.E	Wald	*df*	*p*	Odds ratio	95% CI for odds ratio	
Philantropist	.067	.058	1.364	1	0.243	1.070	−.046	.180
Socialiser	−.050	.036	1.979	1	0.160	.951	−.120	.020
Free Spirit	.162	.068	5.691	1	**<0**.**05**	1.176	.029	.295
Achiever	−.093	.050	3.371	1	0.066	.911	−.192	.006
Disruptor	−.032	.038	.692	1	0.405	.969	−.107	.043
Player	−.040	.037	1.119	1	0.290	.961	−.113	.034

## Discussion

The aim of this study was to validate the preference matrix derived from a prior literature review by assessing whether similar relationships emerge within this research. Either if the relationships of the matrix M1 represented the relations found on the scoping review are found experimentally.

Findings indicate that four mechanisms were selected by more than half of the participants: self-monitoring (*N* = 149), progression (*N* = 127), challenge (*N* = 112), and quest (*N* = 111). No significant associations were identified between these mechanisms and the Hexad Scale dimension except for self-monitoring. Therefore, it can be inferred that these four mechanisms are universally appreciated by participants, regardless of their individual profiles, and should be systematically incorporated into mHealth applications. With the exception of self-monitoring, which could be integrated solely for player users.

### Comparison between preference matrix from the scoping review (M1) and preference matrix based on the results’ study (M2)

Three of the eight significant relationships identified in this study were absent from the original preference matrix M1: the association between players and self-monitoring, free spirits and demonstration of behavior, and achievers and demonstration of behavior.

Therefore, this study contributes to the refinement of the preference matrix M1 by incorporating these three newly identified significant relationships.

**Figure 3.**
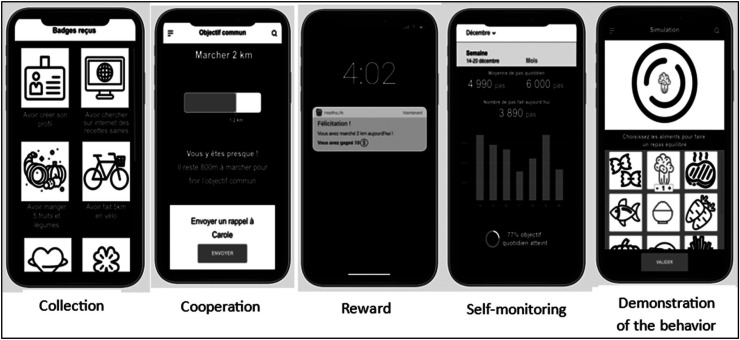
Preferred mechanisms according to user typologies.

In the following section, we provide a more detailed analysis of the five mechanisms that yielded significant regression results. Figure 3 illustrates these five mechanisms, as they were presented to participants in the questionnaire, accompanied by brief descriptions.

### Collection

In our study, the term “collection” denotes an assemblage of items or badges accumulated progressively over time. Our findings indicate player (OR = 1.11, *p* < 0.05) participants exhibited a stronger preference for the collection mechanism compared to other mechanisms and that philanthropist (OR = .77, *p* < 0.01) exhibited an aversion preference for this mechanism.

These results align with existing literature for player but are incongruent for philantropist. Within the preference matrix M1, the collection mechanism was favored by philanthropists,^27,28^ players,^12,21,26,27^ and achievers.^21,27^

The pronounced tendency of player participants to opt for the collection mechanism may be attributed to their motivation by extrinsic rewards, such as badges.

### Cooperation

The cooperation mechanism refers to the collaboration between app users to achieve a common goal, such as walking a predefined distance per day.^33^ Our results indicate that socializer participants selected the cooperation mechanism more frequently than other mechanisms (OR = 1.14, *p* < 0.01) and assigned it a higher motivational score (OR = 1.10, *p* < 0.05).

This finding is strongly supported by five studies in the preference matrix M1.^21,22,24,25,27^ Furthermore, it seem to be consistent with the characteristics of this profile, which describes players who are primarily motivated by interactions with other users.

### Rewards

The reward mechanism involves granting virtual incentives to users upon the successful completion of a targeted action.^33^ Unlike the collection mechanism, rewards do not necessarily belong to a predefined set of collectible items.

Players exhibited a higher tendency to select the reward mechanism (OR = 1.39, *p* < 0.01) and assigned it a greater motivational value (OR = 1.31, *p* < 0.01).

The preference matrix (M1) highlights a preferential association between these participant profiles and the reward mechanism.^12,21,25,27^ This preference may also be attributed to the characteristics of this profile, which is primarily driven by extrinsic rewards.

### Self-monitoring

The self-monitoring mechanism enables users to track their health-related behaviors by providing information about their activities, such as displaying the number of steps taken in a day.^33^

Our results indicate that player participants selected this mechanism less frequently (OR = .88, *p* < 0.05). However, this finding is not supported by the preference matrix M1. Therefore, these results introduce a new entry into the matrix.

### Demonstration of the behavior

The demonstration of behavior mechanism simulate the causes and effects of users’ actions, such as simulating a meal and its impact on their diet.^33^

In our study, free spirit participants selected this mechanism more frequently than other mechanisms (OR = 1.25, *p* < 0.01) and assigned it a higher motivational score (OR = 1.18, *p* < 0.05). These findings are not present in the preference matrix M1, thereby introducing a new relationship into the matrix.

Two profiles, socializers (OR = .92, *p* < 0.05) and achievers (OR = .86, *p* < 0.05) selected this mechanism less frequently than other mechanisms. The lack of preference between this mechanism and socializers contradicts the preference matrix M1, which previously indicated a positive association.^25^ In contrast, the aversion relationship between achievers and this mechanism constitutes a new entry in the preference matrix M1.

### Relations according to profile

This study did not yield significant results for the Disruptor profile. Moreover, the mean participant scores were lowest on this profile (M = 15.22, SD = 14.50), which may partly explain the absence of observable preferences among individuals scoring high in this dimension. Additionally, the Disruptor profile reflects a tendency to challenge the system and push its boundaries. However, none of the mechanisms implemented in our study were designed to elicit or measure such behaviors. A summary of results and preference matrix is presented in Table 14.

**Table 14.** Mechanisms preferred and non-preferred per Hexad-scale dimension (in bold congruent finding between our test and the literature, in italic incongruent).

Hexad scale dimension	Mechanisms preferred	Mechanisms non-preferred	Mechanisms prefered on literatures	Mechanisms non-preferred on literatures
Disruptor			Self-Monitoring, Social Comparison, Progression, Competition, Rewards, Challenge, Avatar, Customization	Self-monitoring, Progression
Philanthropist	**Demonstration of the behavior**	*Collection*	Demonstration of the Behavior, Social support, Progression, Cooperation, Collection, Rewards, Quest, Challenge, Customization	
Socialiser	Collection, **Cooperation**	*Demonstration of the behavior*	Demonstration of the Behavior, Self-monitoring, Punishment, Social comparison, Social support, Progression, Competition, Cooperation, Rewards, Quest, Challenge, Avatar, Customization	Self-monitoring, Progression
Player	**Rewards**, **Collection**	*Self-monitoring*	Punishment, Social comparison, Social support, Progression, Competition, Cooperation, Collection, Rewards, Quest, Challenge, Avatar, Customization	
Free spirit	Demonstration of the behavior		Social Support, Progression, Cooperation, Rewards, Quest, Challenge, Avatar, Customization	
achiever		Demonstration of the behavior	Social comparaison, Progression, Competition, Cooperation, Collection, Rewards, Quest, Challenge, Avatar, Customization	Rewards

### Operationalizing the preference matrix

To apply the preference matrix in real-world settings, two steps are required: first, identifying the user's typologies; second, tailoring the app's features accordingly. While personality assessments typically involve standardized questionnaires, these can be burdensome and may reduce user engagement. Automatic profiling methods, using data such as smartphone usage,^40,41^ social media activity,^42–44^ or wearable data,^45,46^ offer alternatives, though they raise privacy concerns. To balance personalization and user autonomy, we suggest offering this feature as optional, using a short, validated questionnaire when users opt in.

Based on the identified profile, the app would then activate only the mechanisms relevant to the user's dominant traits, as indicated by the matrix. Personalization can target different profiling approaches and can be introduced either during onboarding or later, depending on user choice.

From a policy perspective, the framework can guide the development of mHealth programs that are both evidence-based and user-centered. Prioritizing broadly appealing mechanisms such as self-monitoring and progression can maximize engagement across diverse user groups, while targeted adaptations for underrepresented profiles can help reduce digital health disparities. Scaling such personalization will require compliance with privacy regulations and clear consent processes, but offers the potential to improve both reach and effectiveness of publicly funded digital health interventions.

### Limitations

A number of limitations should be acknowledged. First, the requirement for participants to select exactly five mechanisms out of fifteen may have introduced bias. Some participants may have included mechanisms they did not find particularly motivating simply to meet the selection quota, while others might have been inclined to select more than five if permitted. We adopted a limited-choice format, asking participants to select their five preferred mechanisms, in order to avoid extending the questionnaire's length, a factor known to significantly raise dropout rates.^47,48^ Restricting the selection to five mechanisms also compelled participants to prioritize, thereby reducing common rating biases such as acquiescence, social desirability, or consistency motif.^49^

Second, the sample was not representative of the general population, as it included a disproportionate number of university students, a demographic typically characterized by younger age (*M*_age_ = 29.42, SD_age_ = 10.41). This homogeneity may limit the generalizability of the findings. However, evidence from digital behavior change and web-based health interventions indicates that age does not mediate the observed effects on health behaviors.^50,51^ Evidence from systematic reviews indicates that demographic moderators such as age, sex, and education generally exert weak or inconsistent effects on these techniques.^50^ Furthermore, individuals most likely to use health applications tend to be younger and have higher incomes,^52^ characteristics that align closely with our sample, further supporting the applicability of our results.

Third, although a priori power analysis was conducted, the resulting sample size appears to have been insufficient for detecting effects across the full set of mechanisms. Indeed, significant results were obtained for only 5 out of 14 mechanisms, suggesting that the initial power calculation may need to be revisited in future studies.

Finally, the customization mechanism could not be adequately assessed. Due to its broad and complex nature, it was not feasible to represent this mechanism effectively within the constraints of standard mockup screens.

## Conclusion

This study sought to validate the preference matrix (M1) by examining whether similar preference patterns could be observed in an experimental setting. Four mechanisms, self-monitoring, progression, challenge, and quest, were selected by more than half of participants, suggesting they have broad, cross-profile appeal. These mechanisms should be prioritized for integration in mHealth applications aiming for wide user engagement. Significant associations between user typologies and five mechanisms were identified, with three newly observed relationships, Player–self-monitoring, Free Spirit–demonstration of behavior, and Achiever–demonstration of behavior, not present in the original matrix. These findings contribute to refining and expanding the existing preference model. The Player profile showed the strongest and most consistent associations, aligning with its sensitivity to reward-driven and interactive features. Conversely, no significant associations were found for the Disruptor profile, likely due to both its lower representation and the absence of mechanisms designed to engage system-challenging behaviors. It would be interesting to replicate this study with a larger, less student-dominated panel to get better representation and more significant results on mechanism preference relations by Hexad Scale.

## Supplemental Material

sj-docx-1-dhj-10.1177_20552076251393402 - Supplemental material for Model to personalize mobiles applications according to the gamification user types for health behavioral change Supplemental material, sj-docx-1-dhj-10.1177_20552076251393402 for Model to personalize mobiles applications according to the gamification user types for health behavioral change by Laëtitia Gosetto, Gilles Falquet and Fréderic Ehrler in DIGITAL HEALTH
